# Compromised browning plasticity of primary subcutaneous adipocytes derived from overweight Chinese adults

**DOI:** 10.1186/s13098-020-00599-z

**Published:** 2020-10-22

**Authors:** Yao Qiu, Lizhi Sun, Xiaolin Hu, Xin Zhao, Hongyan Shi, Zhao Liu, Xiao Yin

**Affiliations:** 1 Department of Surgery, Shandong University Affiliated Jinan Central Hospital, Jinan, Shandong People’s Republic of China; 2 Department of Clinical Laboratory, Shandong University Affiliated Jinan Central Hospital, Jinan, Shandong People’s Republic of China; 3 Department of Endocrinology and Metabolism, Shandong University Affiliated Jinan Central Hospital, Linong road 8, 5-1-801, Jinan, 250013 Shandong People’s Republic of China

**Keywords:** Adipocyte browning, Chinese, Norepinephrine, Overweight, Subcutaneous adipose tissue

## Abstract

**Purpose:**

People with obesity have a compromised browning capacity of adipose tissue when faced with sympathetic stimuli. This study aimed to determine whether norepinephrine treatment can enhance the induction of precursor cells from human white adipose tissue to differentiate into adipocytes that express key markers of beige adipocytes, and if there is a difference in this capacity between normal weight and overweight individuals.

**Methods:**

Stromal vascular cells derived from subcutaneous white adipose tissue of normal weight and overweight groups were induced to differentiation, with or without norepinephrine, into adipocytes. Oxygen consumption rate, lipolysis, the expression of uncoupling protein 1 and other thermogenic genes were compared between different adiposity and treatment groups.

**Results:**

Peroxisome proliferator activated receptor γ- coactivator-1 alpha (PGC-1 α) and uncoupling protein 1 gene expression increased significantly in the normal weight group, but not in the overweight group, with norepinephrine treatment. The increments of lipolysis and oxygen consumption rate were also higher in adipocytes from the normal weight group with norepinephrine treatment, as compared with those of the overweight group. PR domain containing protein 16 (PRDM 16) gene expression was higher in the normal weight group compared with that in the overweight group, while there were no significant changes found with norepinephrine treatment in either the normal weight or overweight group.

**Conclusions:**

Adipogenic precursor cells derived from overweight individuals were less prone to differentiate into beige-like adipocytes when facing sympathetic stimuli than normal weight ones, resulting in the compromised sympathetic-induced browning capacity in subcutaneous white adipose tissue in overweight individuals, which occurred before the onset of overt obesity.

## Introduction

Brown adipose tissue (BAT) dissipates energy as heat via the action of uncoupling protein 1 (UCP-1), which is metabolically active in human, and greater human BAT (hBAT) activity is associated with lower adiposity and glycemia, suggesting regulatory links with energy metabolism. Thus, hBAT represents an auspicious perspective for treating metabolic disorders [1, 2]. There is rarely classic brown adipocytes in adipose tissue of human adults, beige adipocytes arise postnatally in white adipose tissue (WAT) in response to certain external cues [3, 4], and UCP-1 positive fat depot can be considered as hBAT [1]. hBAT has emerged as a therapeutic target in obesity, and the strategy of using selective β3-adrenergic receptor (β3-AR) agonists has met with some recent success in healthy individuals [5-8]. However, 18F-fluoro-2-deoxy-d-glucose positron emission tomography computed tomography (18F-FDG PET/CT) scans have revealed that hBAT volume and/or function, at least in response to cold, is reduced in obesity [9]. Additionally, hBAT can be activated via acute, oral administration of sympathomimetic ephedrine in lean, but not obese, individuals [10]. Importantly, it is not presently known whether the relationship between reduced hBAT activity and obesity is correlational or causal in nature. Hence, in the present study, we aimed at elucidating whether compromised adipocyte-browning plasticity occurs at an overweight stage, before the onset of overt obesity, usually the expression of beige adipocytes specific genes and protein was used to evaluate the browning capacity of adipocytes, including beige adipocytes marker protein UCP-1, thermogenic genes Peroxisome proliferator activated receptor γ (PPARγ), PPARγ coactivator-1 alpha (PGC-1α), PR domain containing protein 16 (PRDM16) and specific marker molecular of beige pre-adipocytes early B-cell factor 2 (EBf2) et al. To achieve this aim, we recruited overweight individuals and determined whether norepinephrine treatment could augment the induction of primary precursor cells isolated from human subcutaneous WAT to differentiate in vitro into adipocytes that express key markers of beige adipocytes.

## Materials and methods

### Adipose tissue samples

Abdominal subcutaneous adipose tissue samples [1–2 g] were collected from 50 patients who underwent selected Laparoscopic abdominal surgery, abdominal subcutaneous adipose tissue samples (2–5 g) were collected from 20 patients who underwent selected abdominal open operation. Inclusion criteria consisted of individuals who were 40 to 60 years old and who had a body mass index (BMI) of 18 to 29.9 kg/m^2^. Exclusion criteria consisted of any individuals with cancer, thyroid diseases, diabetes, serious infectious diseases, obesity history or significant weight loss, or those taking concurrent medication likely to influence energy homeostasis, or with smoking and coffee consumption. The patients did not be involved in regular exercise programs during the previous 12 months except daily work and household activity. Height, weight, systolic blood pressure (SBP) and diastolic blood pressure (DBP) were measured before surgery. BMI was calculated as weight (kilograms) divided by height (meters) squared. Participants were divided into the normal-weight group (NW group) [BMI < 25 (kg/m^2^)] and overweight group (OW group) [25 ≤ BMI < 30 (kg/m^2^)] according to BMI, and these groups were matched for age and sex. This study was conducted in accordance with the Declaration of Helsinki and was approved by the local Ethical and Research Committee of Jinan Central Hospital (Shandong, China). Informed consent was obtained from all individual participants included in the study.

### Biochemical analyses

Fasted (8–10 h) blood samples were collected from all participants, enzymatic colorimetric assay was used to measure fasting plasma glucose (FPG) (Hexokinase activity assay, Abcam), total cholesterol (Cholesterol Gen.2, Roche), high density lipoproteins (HDLC) cholesterol (LDL-Cholesterol plus 2nd generation, Roche), triglycerides (Triglycerides/Glycerol Blanked, Roche) concentrations using Dimension Autoanalyzer (Cobas501, Roche, Switzerland). Low-density lipoprotein (LDLC) cholesterol serum concentration was calculated with Friedewald’s formula. Basal Insulin was analyzed by an immunoradiometric assay (BioSource International, Camarillo, CA, USA) in a Beckman Coulter (Fullerton, CA, USA). The homeostatic model assessment for insulin resistance index was calculated using the formula: HOMA-IR = fasting serum insulin (Fins, mU/L) *fasting plasma glucose (FPG, mmol/L) /22.5.

### Isolation of stromal vascular cells (SVCs) from adipose tissue

Adipose tissues were minced and digested with 1 mg/ml collagenase in a shaking water bath at 37℃ for 15–30 min, which were then filtered through a 250-μm mesh. The cell suspension was spun by centrifugation at 2000 rpm for 10 min, and adipose-derived SVCs were then obtained in the pellet. The mature adipocytes and part of the SVCs were stored at − 80℃ for RNA or protein extraction.

### Human primary adipocyte culture

Human SVCs derived from adipose tissue were cultured in DMEM/F-12 medium and grown to confluence. Adipogenic differentiation was induced by addition of a hormone cocktail in media for two days [10% fetal calf serum; isobutylmethylxanthine (Sigma), 0.5 mM; dexamethasone (Sigma), 5 µM; rosiglitazone (Cayman), 1 µM; insulin (Sigma), 5 µM; triiodothyronine (Sigma), 1 nM; indomethacin (Sigma), 125 µM] followed by maintenance media until time of harvest [DMEM/F-12 medium in the presence of rosiglitazone (Cayman), 1 µM; insulin (Sigma), 5 µM; triiodothyronine (Sigma)]. For norepinephrine treatment (NW-NE and OW-NE groups), cells were treated with 1 μM of norepinephrine during adipogenic induction, and were then collected at days 0, 2, 5, 8, 11, and 14 during adipocyte differentiation for RT-PCR analysis.

### Oil Red O staining

Differentiated adipocytes were fixed with 10% formaldehyde at 4 °C for 1 h. After washing with 60% isopropanol, the fixed cells were stained with 0.4% Oil Red O in 3:2 (v/v) isopropanol/H 2 O for 30 min at room temperature and then rinsed three times with water. Lipid accumulation was observed under inverted light ZEISS microscope.

### Quantitative reverse transcriptase PCR (qRT–PCR)

Total RNA was extracted using Trizol reagent (Invitrogen, USA), following the manufacturer’s recommendations. qRT–PCR was performed using a commercial kit (Takara, Japan) and the ABI 7500 Sequence Detection system (Applied Biosystems, USA). Relative mRNA expressions of the indicated genes were calculated using the comparative CT method. Relative UCP-1, PGC-1α, PRDM16 and PPARγ genes expression during adipocyte differentiation was determined by the 2^−ΔΔCt^ method to compare post-differentiation with pre-differentiation values, 18S rRNA levels served as an endogenous control to normalized threshold cycle (CT) value.

### Western blot analysis

Cells were homogenized with RIPA lysis buffer plus protease-inhibitor and protein concentrations were determined by a BCA Protein Assay kit (Beyotime, China). The total of 50 μg of protein from each sample was separated by 10%SDS-PAGE, transferred onto a PVDF membrane, and probed with the following specific primary antibodies: anti-UCP-1 (CST, USA) and anti-β actin (CST, USA), followed by incubation with appropriate secondary anti-rabbit/mouse IgG conjugated with HRP (Proteintech, China) for 1 h at room temperature. Protein bands were detected using Chemiluminescent HRP Substrate (Cell Biosciences, Santa Clara, USA), and band density were analyzed with Image J Analysis Software (NIH). Analyses were conducted as fold-change in protein band density values of the four groups, with OW-con group values normalized to one.

### Cellular respiration

SVCs derives from adipose tissue were directly seeded in the Seahorse plate and induced to differentiation with or without norepinephrine treatment using the methods described before. At day 14 of differentiation, oxygen consumption was measured at 37 °C in cultured fat cells using the Seahorse XF24-3 extracellular flux analyzer (Agilent, USA). Adipocytes were incubated in XF-assay medium containing 25 mM of glucose, 1 mM of sodium pyruvate and 2 mM of l-glutamine (GlutMAX, Gibco). The oxygen consumption rate (OCR) was measured at baseline and followed by sequential stimulation with oligomycin (1 μM, Sigma-Aldrich), carbonyl cyanide-4(trifluoromethoxy)phenylhydrazone (FCCP, 1 μM, Sigma-Aldrich), and antimycin A (2 μM, Sigma-Aldrich). Subtracting antimycin A-insensitive OCR (non-mitochondrial respiration) from baseline OCR reveals basal mitochondrial respiration. Oligomycin was added to block ATP production and, thus, to render uncoupled respiration, and FCCP rates represent maximal respiratory capacity. Experiments were repeated at least 3 times. Basal, uncoupled and maximal respiratory rates were normalized by the total-cell protein concentration using a BCA Protein Assay Kit (Beyotime, China).

### Lipolysis assay

For determination of norepinephrine-induced lipolysis, fresh media and treatments (PBS vehicle or 1 μM of norepinephrine) were added to differentiated adipocytes, which were left for 6 h prior to collection of the media for determination of glycerol concentration. The glycerol concentration in the media was quantified using a commercially available EnzyChrom™ Adipolysis Assay Kit (Basbiotech, China), according to manufacturer’s instructions, and was normalized by the total-cell protein concentration using a BCA Protein Assay Kit (Beyotime, China).

### Statistical analysis

All the data generated in this study met the normal distribution except the fasting insulin which was log transformated because of skew distribution. The quantitative physical characteristics and clinical measurements, the EBf2 gene expression levels of the NW and OW groups were analyzed by the Student’s t-tests; the gender difference between the NW and OW groups were analyzed by X^2^ test. In the NE treatment group, the difference of UCP-1 and other thermogenic biomarkers between the NW and OW groups were analyzed by paired two-tailed Student’s t-tests, the same statistical analysis was used in the control groups. Comparisons between pre- and post-treatment data within the same treatment group were analyzed by analyses of variances (ANOVA). The calculations were performed by SPSS for Windows (version 18.0 software; IBM Corporation, Chicago, IL, USA). Results were expressed as mean ± SD, and differences between groups were considered statistically significant at P < 0.05.

## Results

### The characteristics of participants

The anthropological and clinical features of the participants were showed in Table 1. SVCs derived from 50 adipose-tissue samples were used for EBf2 gene expression analysis, and those derived from another 20 adipose-tissue samples were used for primary adipocyte cultures. EBf2 gene analysis and primary adipocytes culture were carried out with independent sample groups due to the limitation of the adipose tissue volume we could get from the operation (Additional file 1). The participants were divided into NW and OW groups according to their BMI as described in methods, the two groups were matched for age and sex. The fasting serum insulin, HOMA-IR index and blood pressure was higher in the OW group compared to those in the NW group. There was no significant difference in total cholesterol, low density lipoprotein cholesterol, high density lipoprotein cholesterol and triglyceride levels between the OW and NW groups.

**Table 1:** The clinical characteristics of participants

	EBf2 gene analysis		Primary cell culture	
	NW group	OW group	NW group	OW group
N	25	25	10	10
Gender (M/F)	13/12	14/11	5/5	5/5
Age (years)	53.64 ± 12.83	52.40 ± 11.00	52.80 ± 8.46	51.40 ± 9.55
BMI (kg/m^2^)	22.47 ± 2.02	27.68 ± 1.57^#^	23.28 ± 1.50	27.68 ± 1.17^#^
DBP (mm Hg)	76.92 ± 10.48	84.28 ± 6.69^#^	84.40 ± 12.70	89.80 ± 7.98
SBP (mm Hg)	123.84 ± 13.90	135.60 ± 19.24^#^	132.60 ± 22.21	142.20 ± 22.66^#^
FPG (mmol/L)	4.86 ± 0.41	4.92 ± 0.34	5.08 ± 0.39	4.96 ± 0.45
Fins (mIU/L)	5.29 ± 2.5	8.27 ± 3.42^#^	4.39 ± 2.07	8.38 ± 2.56^#^
HOMA-IR	1.14 ± 0.56	1.82 ± 0.85^#^	0.99 ± 0.51	1.84 ± 0.64^#^
TC (mmol/L)	4.67 ± 0.39	4.82 ± 0.45	5.06 ± 0.42	5.13 ± 0.45
HDLC (mmol/L)	1.03 ± 0.12	1.05 ± 0.15	1.02 ± 0.11	1.03 ± 0.09
LDLC (mmol/L)	2.40 ± 0.86	2.78 ± 0.74	1.97 ± 0.64	2.70 ± 0.47
TG (mmol/L)	1.17 ± 0.45	1.38 ± 0.71	1.03 ± 0.37	1.40 ± 0.27

Values are shown as mean ± SD; The quantitative physical characteristics and clinical measurements of normal weight (NW) and overweight (OW) groups were analyzed by unpaired two-tailed Student’s t-tests; the gender difference between the NW and OW groups were analyzed by X^2^ test. ^#^ P < 0.05 NW vs OW group

*BMI* body mass index, *DBP* diastolic blood pressure, *SBP* systolic blood pressure, *Fins* fasting insulin, *HOMA-IR* Homeostasis model assessment for insulin resistance index, *TC* total cholesterol, *LDLC* low density lipoprotein cholesterol, *HDLC* high density lipoprotein cholesterol, *TG* triglyceride

### Gene-expression analysis of early EBf2 in adipose-derived stromal vascular cells (SVCs)

**Fig. 1:**
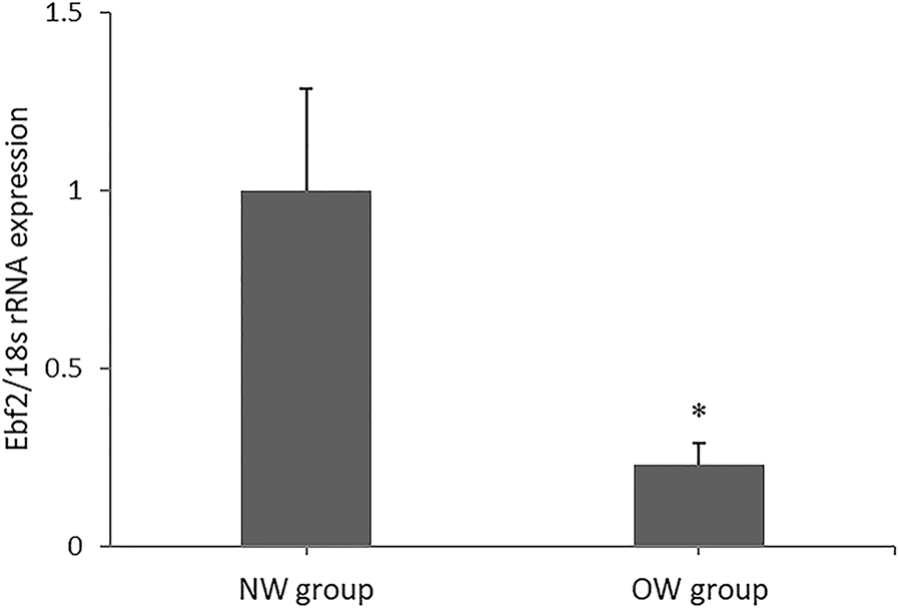
EBf2 expression in adipose-derived vascular stromal cells. Fold change in EBf2 gene expression of vascular stromal cells derived from adipose tissue in the normal weight (NW) and overweight (OW) groups (Values were mean ± SD; NW group expression level normalized to 1.0; n = 25 for the NW and OW group; experiments were repeated three times for each sample; *P < 0.05 NW vs OW group). *NW group* normal weight group, *OW group* overweight group

The EBf2 gene is considered to be a specific marker that regulates molecular profiles of brown and beige pre-adipocytes. Results of the present study showed that the EBf2 mRNA expression in SVCs from subcutaneous adipose tissue of the OW group was significantly lower compared to that of the NW group (P = 0.031; Fig. 1).

### Thermogenic genes expression during adipocyte differentiation and the effect of norepinephrine treatment

**Fig. 2:**
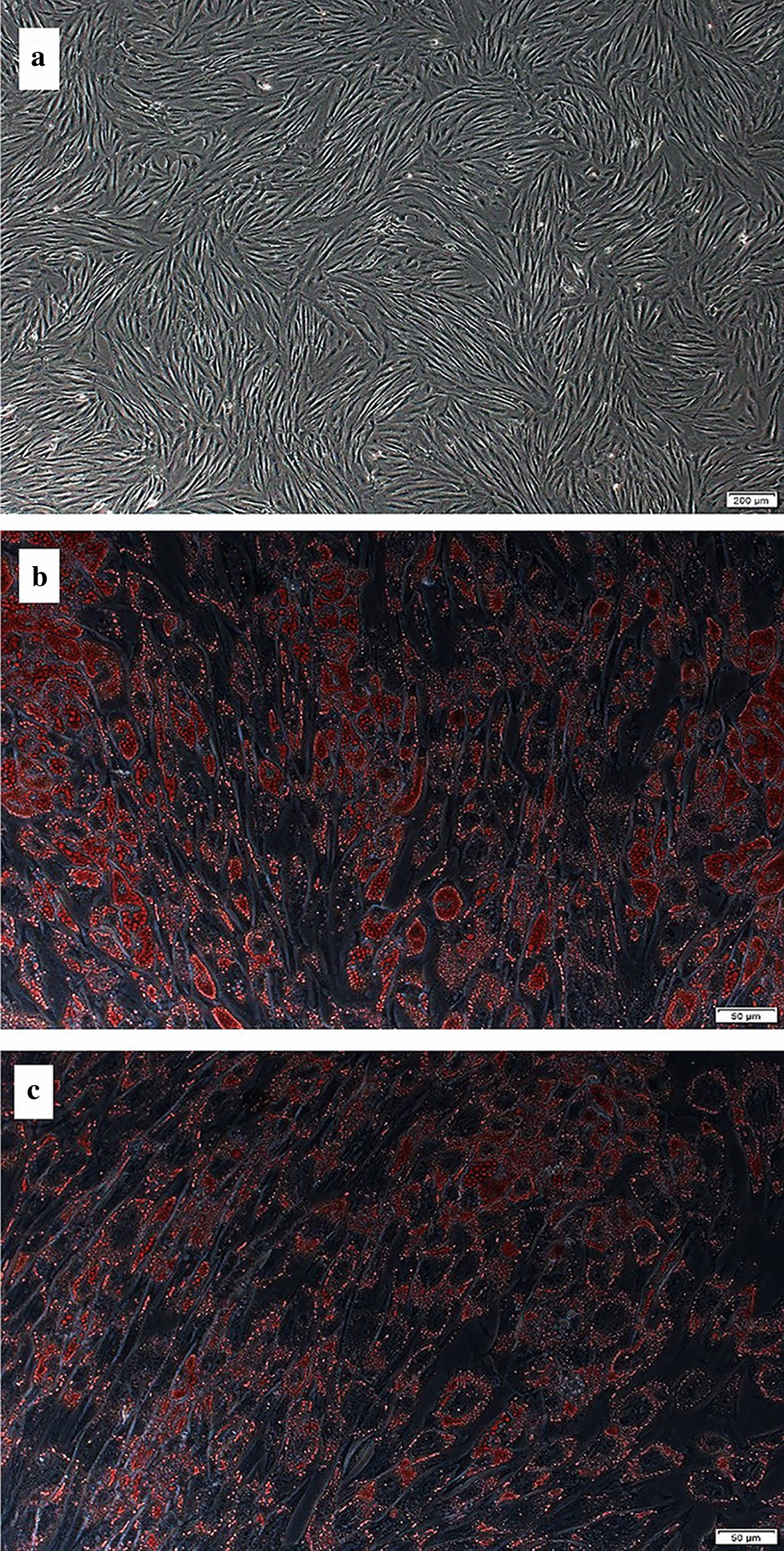
Oil-red O-staining of differentiated adipocytes (day 14). Before (**a**) and after differentiation cells (**b** normal weight group; **c** overweight group) were fixed and stained with oil red-O; images shown are 20 × magnification

The SVCs derived from subcutaneous adipose tissue of NW and OW groups were cultured and treated with standard adipogenic inducers to analyze their differentiation potential. Fold changes of PPARγ gene expression were significantly higher after differentiation compared with that of pre-differentiation in all the four groups (NW-con 8.79 ± 0.94, NW-NE 8.46 ± 0.76, OW-con 8.61 ± 0.92, OW-NE 9.13 ± 0.94, with pre-differentiation normalized to 1.0, P < 0.05), but there was no significant difference between the groups either before or after differentiation. PPARγ gene expression was representative of general adipogenesis; this result indicated that cells from all the four groups differentiated into adipocytes, which was also revealed by the Oil-Red O-staining for lipids (Fig. 2).

**Fig. 3:**
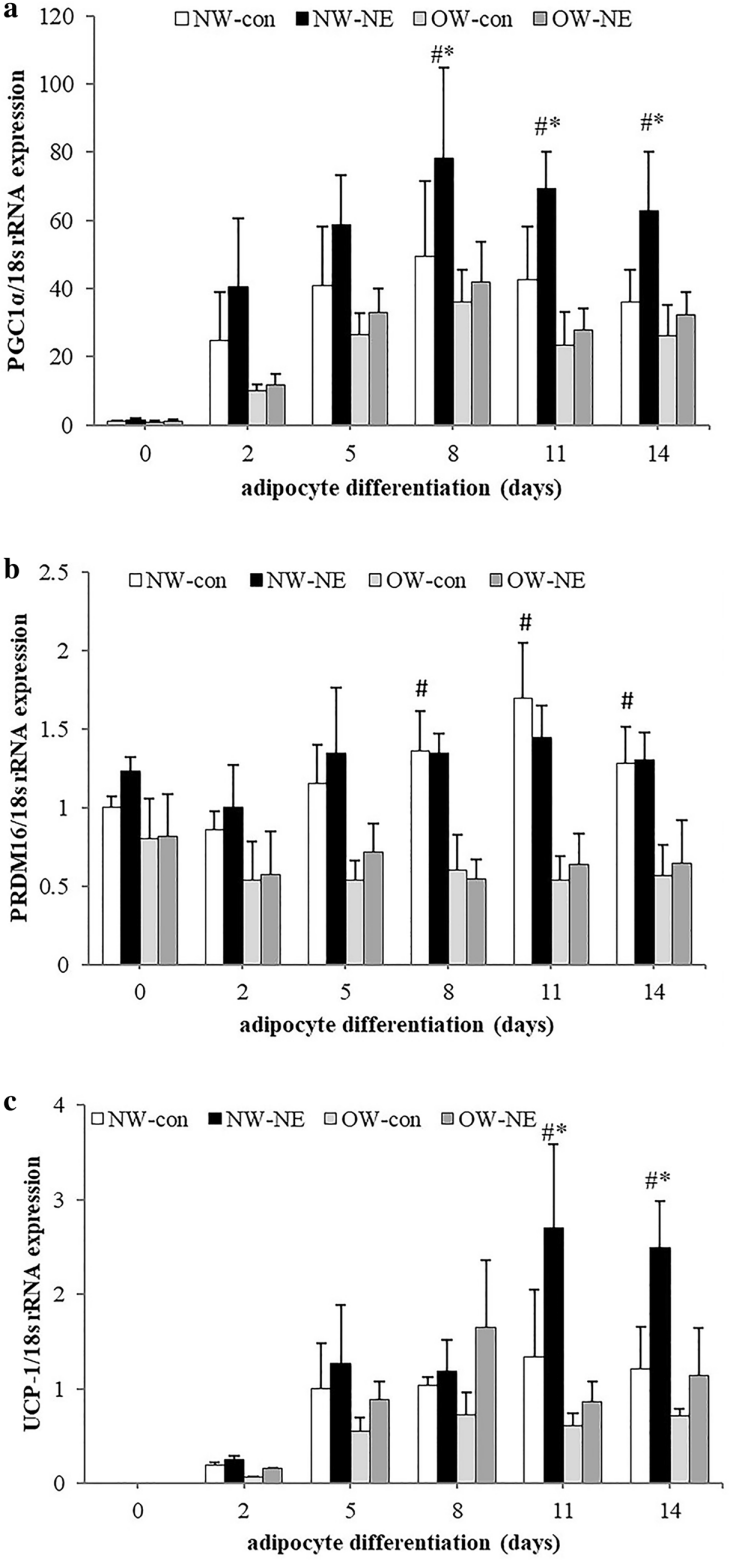
Fold changes of indicated genes during adipocyte differentiation. Normal-weight (NW) compared to the overweight (OW) group and norepinephrine (NE) treatment compared to control (con) group. PGC-1α (**a**) and PRDM16 (**b**) gene expression in the NW-con group at day 0 was normalized to 1, and UCP-1 gene expression (**c**) in the NW-con group at day 5 was normalized to 1. (Values were mean ± SD; n = 10 for the NW and OW group; experiments were repeated three times for each sample; ^#^P < 0.05 NW vs OW group; *P < 0.05 control vs NE treatment). *NW-con* normal weight control group, *NW-NE* normal weight norepinephrine treatment group, *OW-con* overweight control group, *OW-NE* overweight norepinephrine treatment group

We measured the expression levels of beige-adipocyte-marker genes during adipocyte differentiation using norepinephrine treatment to mimic the cold exposure in vitro when indicated. The results showed that the expression of PGC-1α, the key factor in mitochondrial biogenesis, started to increase at day 2 and reached to peak at day 8 during adipocyte differentiation; there was no difference in PGC-1α gene expression between NW and OW groups at each differentiation day. In response to norepinephrine treatment, PGC-1α expression was significantly higher (P < 0.05) compared to that of the control at day 8, 11 and 14 during adipocyte differentiation in the NW group, but there was no significant difference in the OW group with norepinephrine treatment (Fig. 3a).

The gene expression of PRDM 16, the key browning transcriptional regulator, started to increase at day 5 and reached to peak at day 11 during adipocyte differentiation in the NW group, but we did not see such a statistically significant increase in cultured cells from the OW group. The levels of PRDM16 gene expression were significantly lower (P < 0.05) in the OW group compared with those in the NW group at days 8, 11 and 14 during adipocyte differentiation. There was no significant difference in PRDM16 expression with or without norepinephrine treatment in both the NW and OW groups (Fig. 3b).

UCP-1, the marker gene of brown and beige adipocytes, was undetectable at the early stage of adipocyte differentiation; then UCP-1 expression started to become detectable at day 5 and exhibited a 1.5-fold increase at day 11 in the NW group, but there was no significant difference between NW and OW groups in terms of UCP-1 expression during differentiation. With norepinephrine treatment, UCP-1 expressions in the NW group reached a peak at day 8 and increased by 2.4-fold compared with control, and this expression was significantly higher (P < 0.05) at day 8, 11 and 14 during adipocyte differentiation compared to that in both control and OW group. No significant difference was found in terms of UCP-1 expression in the OW group with or without norepinephrine treatment (Fig. 3c).

### UCP-1 protein expression in differentiated adipocytes and the effect of norepinephrine treatment

**Fig. 4:**
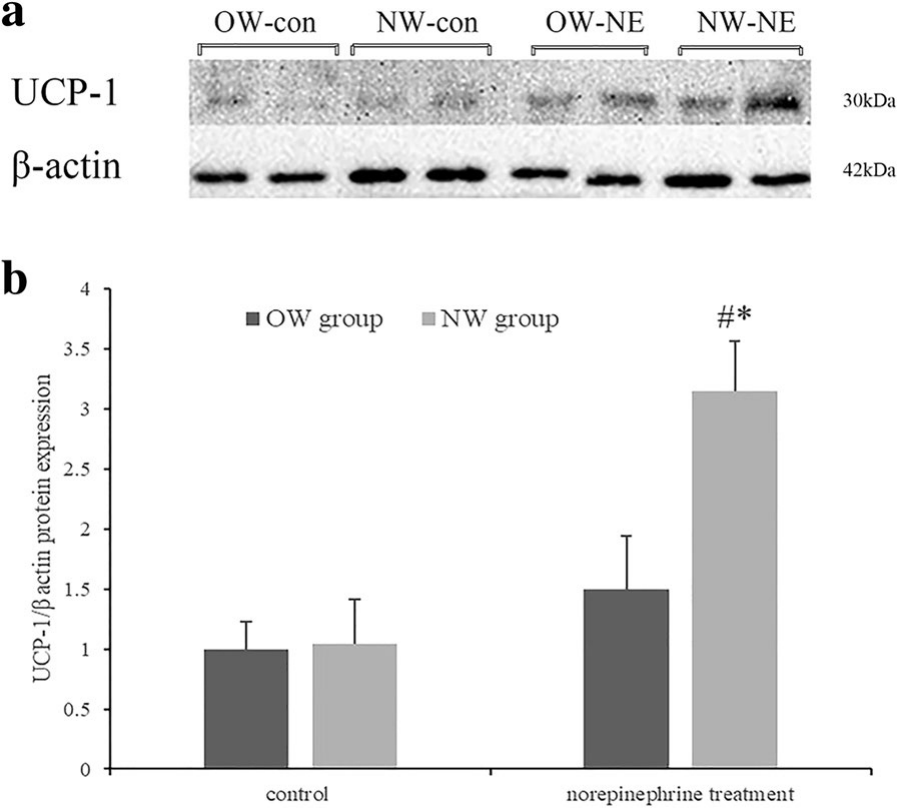
UCP-1 protein content of differentiated adipocytes. Western-blot analysis of UCP-1 protein expression (**a**) and fold-change (**b**) in protein band density values of normal weight group (NW) and overweight group (OW) with (NE) and without (control) norepinephrine treatment, OW-con group values normalized to 1.0 (Values were mean ± SD; n = 10 for the NW-NE, NW-con, OW-NE and OW-con group; ^#^P < 0.05 NW vs OW group; *P < 0.05 control vs NE treatment).*OW-con* overweight control group, *NW-con* normal weight control group, *OW-NE* overweight norepinephrine treatment group, *NW-NE* normal weight norepinephrine treatment group

The differentiated adipocytes of both the NW and OW groups without norepinephrine treatment were used to detect UCP-1 protein expression (Fig. 4), which was similar between the two groups (P > 0.05); this result was in accordance with the UCP-1 gene expression levels of primary mature adipocytes from the NW and OW groups (P > 0.05, data not showed). With norepinephrine treatment, adipocytes from the NW group showed a significant increase in UCP-1 protein expression compared to that of the control group (0.78 ± 0.21 vs 2.36 ± 0.28, P < 0.05). Adipocytes from the OW group also showed a trend of increase in UCP-1 protein expression after norepinephrine treatment, but the difference between the treatment and control group was not statistically significant (P > 0.05).

### Functional analysis of mitochondrial respiration in differentiated adipocytes

**Fig. 5:**
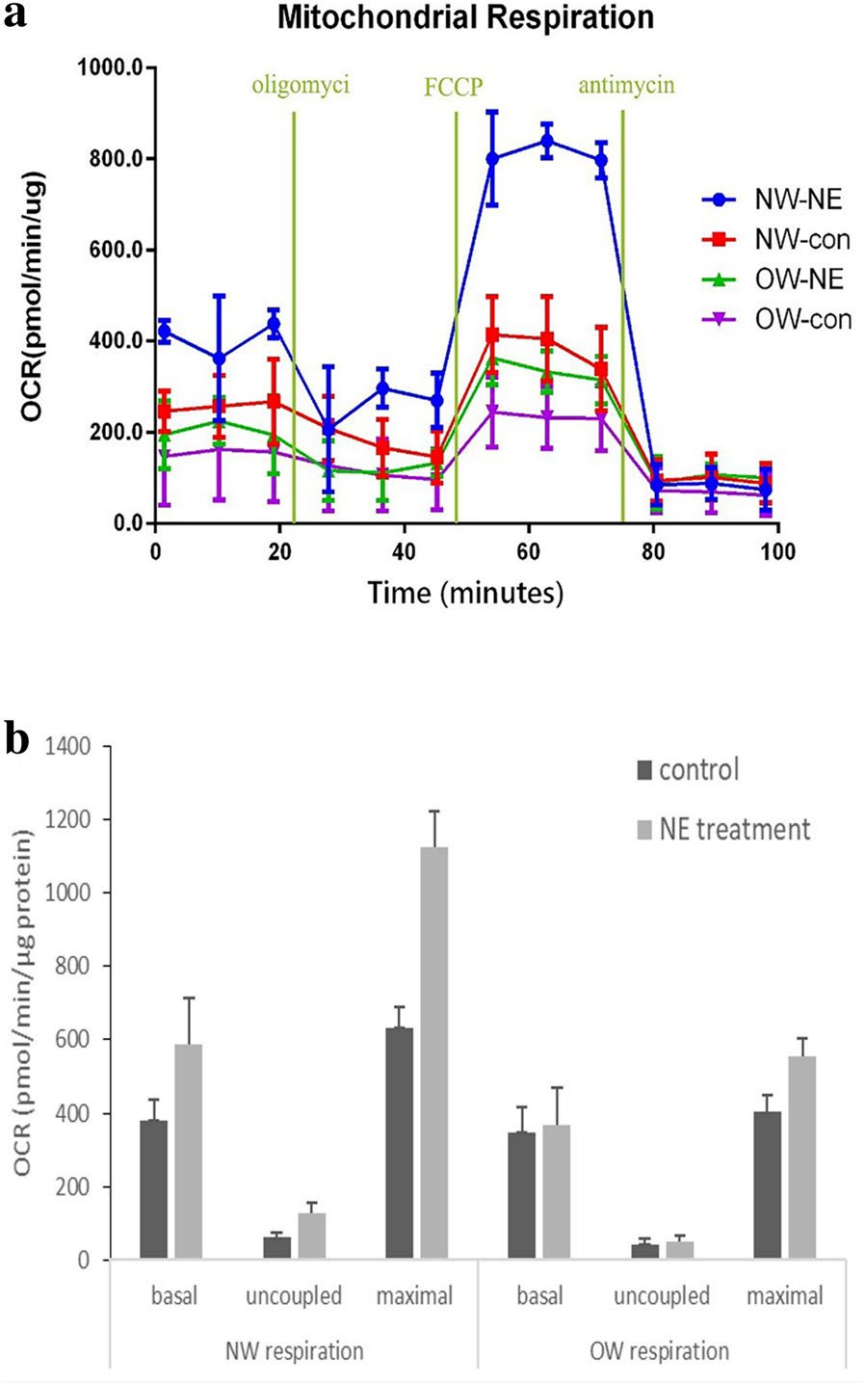
Oxygen consumption rate in differentiated adipocytes. The differentiated adipocytes derived from the normal weight (NW) and overweight (OW) groups treated with (NW-NE and OW-NE) or without (NW-con and OW-con) norepinephrine were used to detect oxygen consumption rate (OCR). The oxygen consumption rate (OCR) was measured at baseline and followed by sequential stimulation with oligomycin, FCCP, and antimycin A. Subtracting antimycin A-insensitive OCR (non-mitochondrial respiration) from baseline OCR reveals basal OCR, oligomycin was added to render uncoupled OCR, and FCCP rates represent maximal respiratory capacity, three data points were collected from each period, and mean value of the three points were calculated for data analysis. Experiments were repeated at least 3 times. **a** Basal, uncoupled and maximal OCR values were normalized by the total-cell protein concentration and compared between different groups (**b**). (Values were mean ± SD; n = 10 for the NW-NE, NW-con, OW-NE and OW-con groups; data used for analysis were mean values of four replicate wells for each sample. ^#^P < 0.05 NW vs OW group; *P < 0.05 control vs NE treatment). *NW-NE* normal weight norepinephrine treatment group, *NW-con* normal weight control group, *OW-NE* overweight norepinephrine treatment group, *OW-con* overweight control group, *OCR* oxygen consumption rate

Mitochondrial respiration is central to adaptive thermogenesis. To assay the functional characteristics of fat cells from individuals of different adiposity, we investigated the mitochondrial oxygen consumption rate (OCR) of differentiated adipocytes treated with (NE) or without (control) norepinephrine derived from NW and OW groups (Fig. 5). At a functional level, the basal, uncoupled and maximal respiratory OCRs of differentiated adipocytes from the OW and NW group were not significantly different (P > 0.05). Without NE stimulation, uncoupled respiration contributed to 16% of the basal respiration rate in the NW group and 14% in the OW group. In the presence of NE, the basal OCR (366.13 ± 57.53 vs 587.63 ± 124.8 pmol/min/μg protein, P < 0.05), uncoupled respiratory OCR (60.59 ± 12.7 vs 125.7 ± 31.2 pmol/min/μg protein, P < 0.05), and maximal OCR (631.24 ± 56.23 vs 1123.34 ± 98.14 pmol/min/μg protein, P < 0.05) went up nearly 1.2–2.0 fold in fat cells from the NW group compared to a minor basal and uncoupled OCR increase in the fat cells from the OW group (P > 0.05). The uncoupled respiration contribution increased to 21% of their basal respiration rate in the NW group in the presence of NE, which was significantly higher than the relative contribution of uncoupled respiration in the cells from OW group (P < 0.05). With NE treatment, the basal OCR (379.80 ± 103.6 vs 587.6 ± 124.8 pmol/min/μg protein, P < 0.05), uncoupled OCR (49.12 ± 17.1 vs 125.7 ± 31.2 pmol/min/μg protein, P < 0.05) and maximal OCR (556.42 ± 46.34 vs 1123.34 ± 98.14 pmol/min/μg protein, P < 0.05) in the fat cells from the OW group were significantly lower than those from the NW group. These data indicated that adipocytes from the NW group were more norepinephrine-responsive, showed a higher respiratory capacity, and exhibited increased uncoupled respiration compared to that from fat cells in the OW group.

### Lipolysis analysis of differentiated adipocytes

**Fig. 6:**
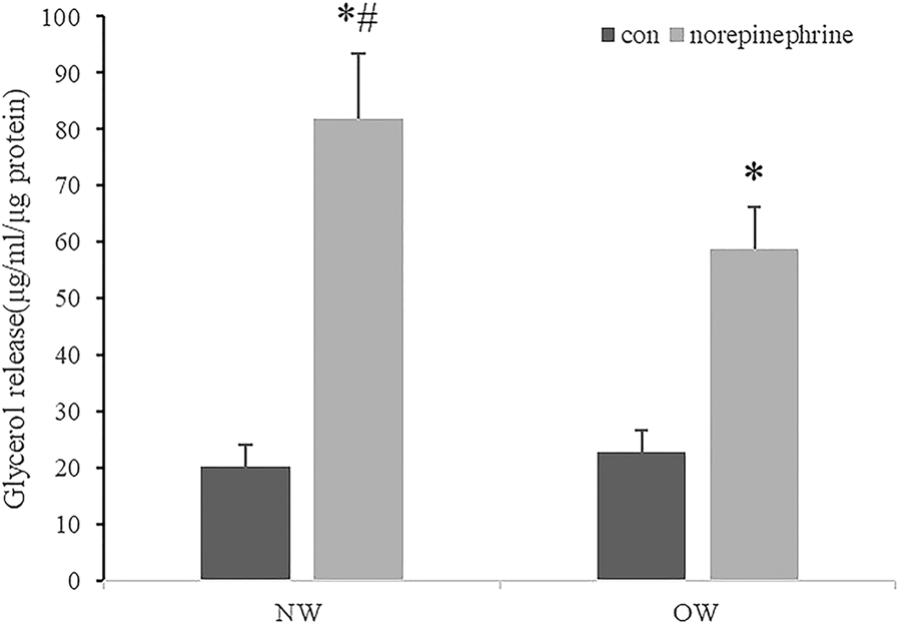
Lipolysis analysis of differentiated adipocytes. Glycerol concentration was measured in the media to determine rates of lipolysis after differentiated cells derived from the normal weight (NW) and overweight (OW) groups were treated with PBS (NW-con and OW-con) or 1 μM norepinephrine (NW-NE and NW-NE) for 6 h (Values were mean ± SD; n = 10 for NW-NE, NW-con, OW-NE and OW-con groups; data used for analysis were mean values of three replicate experiments for each sample; ^#^P < 0.05 NW vs OW group; *P < 0.05 con vs NE treatment). *NW* normal weight group, *OW* overweight group

Lipolysis, measured as glycerol release rate, was not significantly different between the NW and OW groups at basal conditions. However, a significant increase in the lipolysis rate was found in both NW and OW groups (P < 0.05) after norepinephrine (1 μM) treatment for 6 h, and the increment was significantly higher in the NW group compared to the OW group (82.01 ± 11.55 vs 58.8 ± 7.45 μg/ml/μg protein, P < 0.05; Fig. 6).

## Discussion

Brown, beige, and white adipocytes arise from distinct developmental lineages and have different molecular signatures, as analyzed by RNA-seq analysis [3]. EBf2 has been identified as a specific marker gene of brown/beige pre-adipocytes, and activation of EBf2 is likely to be an early step in brown/beige adipose-lineage commitment [11]. The results from the present study showed that EBf2 gene expression was higher in SVCs derived from subcutaneous adipose tissue of normal weight individuals, which implied that the cellular fate of beige versus white adipocytes is largely determined at the precursor cell stage in adult humans.

In the prevailing model, cold temperatures trigger sympathetic discharge, which results in the release of noradrenaline in brown and white adipose tissue that activates β3-AR. Noradrenergic signaling induces the expression of thermogenic genes, such as PGC-1a and UCP-1, in brown adipocytes. β3-AR agonists can stimulate human BAT activity in normal weight adults [5], but this ability was impaired in people with obesity [10] tested via 18F-FDG PET/CT detection. Human subcutaneous WAT cells can be induced to attain BAT characteristics, but this capacity is also reduced in WAT cells from obese individuals [12]. A study we published recently showed that β3-AR protein expression of SVCs and mature adipocytes from overweight individuals was lower compared to that of normal-weight individuals, which suggests that a deficit in adipose-tissue adrenergic signaling occurs before the onset of overt obesity [13].

Most of the data regarding hBAT activity come from populations with obesity [9, 10, 14], which can not determine the causal relationship between inactive BAT and obesity (i.e., whether defective BAT contributes to obesity or vice-versa). Hence, the present study aimed to detect the browning capacity of subcutaneous WAT in overweight individuals and explore if the sympathetic-induced browning potential of subcutaneous WAT changes at a pre-obesity stage.

Exposure to cold temperatures is likely to continue to be an approach for researchers to maximally activate hBAT for measurement of its volume in humans [15]. However, differences in the cooling goals affect the degree of hBAT activation and, therefore, the ability to detect it [16]. Glucose uptake measured by 18F-FDG-PET likely underestimates hBAT activity under physiological conditions, therefore, is informative but not sufficient [15, 16]. Due to the low brown-to-white adipocyte ratio in humans, we believe that adipocyte biomarkers are more useful than imaging approaches to detect beige fat cells that are induced in subcutaneous white-fat depots. Therefore, in the present study, we detected the expression of thermogenesis molecule biomarkers during adipocyte differentiation to elucidate the difference in beige inducible potential of subcutaneous WAT SVCs between lean and overweight individuals.

A defining feature of brown/beige adipocytes is their abundant mitochondria and associated high rate of cellular respiration; cellular mitochondrial content can increase through biogenesis and can be promoted by PGC-1α, which is considered to be a key thermogenic factor [17–19]. Our results showed no difference in the gene expression of this beige-adipocyte biomarker and its multilocular appearance during primary adipocyte differentiation between overweight and lean individuals. However, PGC-1α and UCP-1 gene expression in differentiated adipocytes from lean individuals increased dramatically with the treatment of norepinephrine, but we did not see this increment in the overweight group, which may be due to the reduce β3-AR expression in Chinese overweight individuals [13]. PRDM16 activates a broad program of brown-fat differentiation and functions as a dominant regulatory factor to promote a brown-fat lineage [20–22]. We found that the PRDM16 gene expression in adipocytes from lean individuals showed a two-fold enrichment relative to that from overweight individuals during adipocyte differentiation, but its expression was not affected by norepinephrine treatment in either normal-weight or overweight groups, which is in accordance with a previous study in mice [17]. Our findings indicated that PRDM16 expression was linked to the determination of beige/brown fat-cell differentiation, but not to adaptive thermogenesis.

We also measured the adipocyte oxygen consumption rate to assay the functional difference of differentiated adipocytes from overweight and normal-weight individuals and the consequence of norepinephrine treatment. The data revealed that the mitochondrial uncoupled oxygen consumption was enhanced in differentiated adipocytes with norepinephrine treatment, and the degree of increment was substantially higher in adipocytes from normal weight than that from overweight individuals, the expression level of beige adipocytes marker protein, UCP-1, was in accordance with the results of OCR analysis. This part results indicated that sympathetic stimulation could active the thermogenic capacity of adipocytes from normal weight individuals by increment of UCP-1 gene and protein expression, which is the characteristic of beige adipocytes, but this increased browning capacity facing sympathetic stimuli was impaired in adipocytes from overweight individuals. And norepinephrine intervention was carried out during the period that SVCs were induced to differentiate into adipocytes, which means sympathetic stimuli acting through the β3-AR could promote the beige-like adipocytes differentiation in normal weight individuals, but not so obvious in overweight ones.

Lipolysis analysis of differentiated adipocytes also revealed that the fat cells from normal weight individuals had a higher level of lipolysis when treated with norepinephrine; the results of this catabolic intervention were also observed in a previous human study [5, 23]. Some researchers have recently found that lipolysis in WAT triggers insulin release, which is essential for the replenishment of BAT energy stores and for efficient adaptive thermogenesis [24].

## Conclusions

The present study indicated that SVCs from subcutaneous WAT can be induced to express some biomarkers of beige adipocytes, and cells from overweight individuals showed a lower beige inducible ability compared to that of normal weight individuals. Sympathetic stimuli could enhance the browning plasticity during adipocyte differentiation in SVCs derived from normal weight individuals, but not in overweight individuals. Because most previous findings comparing the browning capacity of human adipose tissue from individuals with different adiposity came from normal weight and obese populations, we have demonstrated that the browning plasticity of subcutaneous WAT changes at an overweight stage, before the onset of overt obesity. Regardless of whether defective BAT is a cause or a consequence of obesity, BAT-activation strategies remain as a potential therapeutic target in overweight and obese individuals. Therefore, discovery of therapeutic agents to activate BAT through specific sympathetic pathways could provide a promising approach for treating obesity in the future.

## Supplementary information

**Additional file 1: Table S1.** Clinical infromation of EBf2 gene analysis group andprimary adipoycte culture group.

## Data Availability

The datasets during and/or analysed during the current study available from the corresponding author on reasonable request.

## Abbreviations

PGC-1α: Peroxisome proliferator activated receptor γ- coactivator-1 alpha
PRDM 16: PR domain containing protein 16
BAT: Brown adipose tissue
UCP-1: Uncoupling protein 1
β3-AR: β3-Adrenergic receptor
18F-FDG PET/CT: 18F-fluoro-2-deoxy-d-glucose positron emission tomography computed tomography
PPARγ: Peroxisome proliferator activated receptor γ
EBf2: Early B-cell factor 2
BMI: Body mass index
FPG: Fasting plasma glucose
HDL: High density lipoproteins cholesterol
LDL: Low-density lipoprotein cholesterol
SVCs: Stromal vascular cells
NE: Norepinephrine
qRT–PCR: Quantitative reverse transcriptase PCR
